# Clinical significance of 4 patients with chronic hepatitis B achieving HBsAg clearance by treated with pegylated interferon alpha-2a for less than 1 year: a short report

**DOI:** 10.1186/1743-422X-6-97

**Published:** 2009-07-08

**Authors:** Jin Yang, Lian-san Zhao

**Affiliations:** 1 Department of Infectious Diseases, Nanshan Affiliated Hospital of Guangdong Medical College, Shenzhen 518052, China; 2 Center of Infectious Diseases, National Key Laboratory of Biotherapy for Human Diseases, West China Hospital, Sichuan University, Chengdu 610041, Sichuan Province, China

## Abstract

We report 4 chinese patients with hepatitis B e antigen-positive chronic hepatitis B achieving clearance of HBsAg by using pegylated interferon alpha-2a for less than 1 year, to provide one clinical clue for the treatment of chronic hepatitis B.

## Background

Chronic infection with Hepatitis B virus(HBV) is a major global health problem, affecting 350–400 million people worldwide [1,2]. Patients who have HBV infection with positivity for hepatitis B e antigen(HBeAg) and persistently active disease have increased risks for progressive disease leading to liver cirrhosis and hepatocellular carcinoma[3]. Therefore, for patients with HBeAg positive chronic hepatitis B(CHB), anti-viral therapy is most important if they have indications for treatment. The end points to assess efficacy of therapy include: reduction of serum HBV DNA to the undetectable level, normalization of alanine aminotransferase(ALT) levels, HBeAg seroconversion and an improvement of liver disease at histology[4]. But it is gradually thought that the ultimate therapeutic goal of anti-HBV therapy should furthermore make patients achieve clearance of hepatitis B surface antigen(HBsAg) or even HBsAg seroconversion [5,6]. In view of current anti-HBV drugs, however, it is extremely difficult to implement this goal. Nevertheless, on condition that there is a treatment with effective anti-viral drugs and appropriate therapeutic schemes, some patients may still be able to achieve it.

## Methods

### Patients

All 4 patients aged from 14 to 49 yrs-old, including 3 males and 1 female, had suffered from CHB for 1 to 3 years with the sera profile of HBeAg, HBsAg positive, and HBV DNA leveled from 2.71 × 10^6 ^copies/mL to 5.00 × 10^7 ^copies/mL. They came to our hospital for pegylated interferon alpha-2a(Pegasys) treatment from March 2006 to Ten 2007. All of them had neither complications nor other accompanying diseases, also with no history of anti-HBV drug use.

### Methods of treatment

#### Classification of methods

Pegasys monotherapy Case 1 was treated with pegasys at a dose of 135 ug intracutaneously one time per week(for her age was below 18). Case 2 was treated with pegasys at a dose of 180 ug intracutaneously one time per week.

Sequential therapy As both leucopenia and thrombocytopenia occurred in case 3 and case 4 during the treatment of pegasys, we adopted sequential therapy with pegasys and entecavir(Baraclude). Pegasys was administered at a dose of 180 ug intracutaneously one time per week. Baraclude was administered at a dose of 0.5 mg orally one time per day.

#### Periodic monitoring of anti-viral therapy

Leukocyte and platelet counts, serum HBV DNA and ALT were detected once every month. HBV markers(HBsAg and anti-HBs, HBeAg and anti-HBe, anti-HBc) were detected once every three months. Among them, serum HBV DNA was arrayed by fluorescent quantitative PCR and HBV markers were arrayed by ELISA.

## Results

### Therapeutic efficacy

Within less than 1 year, serum HBV DNA loss, ALT normalization, HBeAg seroconversion, HBsAg clearance occurred in the 4 patients treated with either pegasys monotherapy (the longest period of treatment was 40 weeks, the shortest period of treatment was 20 weeks) or sequential therapy with pegasys and baraclude (the longest period of treatment with pegasys was 24 weeks, the shortest period of treatment with pegasys was 16 weeks). Among them, the latest time when HBsAg clearance occurred (in case 4) was at week 44, and the earliest time when HBsAg clearance occurred (in case 1) was at week 17. Furthermore, HBsAg seroconversation occurred in case 1 at week 20, which is sustained positive in 48 weeks follow-up, while HBsAg negative also kept steady in case 2, case 3, case 4 in 60 weeks follow-up, 60 weeks follow-up, 72 weeks follow-up, respectively. All of them are kept under observation until now. The Variations of sera HBV DNA, ALT and HBV markers in the four patients are outlined in Table 1.

**Table 1:** Therapeutic efficacy of pegasys in 4 patients with chronic hepatitis B

**Characteristics**	**Case 1**	**Case 2**	**Case 3**	**Case 4**
Sex	Female	Male	Male	Male
Age(yr)	14	24	39	49
Course of disease^a^(yr)	3	2	1	1
Therapy	Monotherapy	Monotherapy	Sequential therapy	Sequential therapy
Course of treatment^b^(wk)	36	40	24	44
HBV DNA undetectable^c^	Week 14	Week 16	Week 20	Week 14
ALT normalization	Week 36	Week 26	Week 23	Week 14
HBeAg seroconversion	Week 16	Week 22	Week 23	Week 38
HBsAg clearance	Week 17	Week 40	Week 24	Week 44
HBsAg seroconversion	Week 20	--	--	--

yr, year; wk, week; ALT, alanine aminotransferase; HBeAg, hepatitis B e antigen; HBsAg, hepatitis B surface antigen; HBV, hepatitis B virus.

^a ^interval between the first time when HBsAg-positive was detected and the date before treatment.

^b ^cumulate course between the first day of anti-HBV treatment and the date when complete response occurred.

^c ^HBV DNA<1000 copies/ml.

### Adverse events

During the course of pegasys treatment, apparent reduction of leukocyte and platelet counts had occurred in 2 patients (case 3 and case 4) at week 4 and week 10 of therapy respectively, but returned to baseline levels one month after sequential therapy was adopted (pegasys was paused temporarily, baraclude was administered until the leukocyte and platelet counts recovered). There were no other remarkable adverse events in all the four cases during the total course of anti-viral therapy.

## Discussion

The aims of treatment for CHB are to achieve sustained suppression of HBV replication, thereby inducing remission of liver diseases and interrupting progression to liver cirrhosis or hepatocellular carcinoma[7]. Pegylated interferon alpha-2a is created by attaching a large, branched, 40-kD polyethylene glycol molecule to interferon alfa-2a[8], This allows for convenient once-weekly dosing, with effective serum levels maintained throughout the dosing interval[9]. It could efficaciously inhibit the replication of HBV and help eradicate HBV infectious hepatocytes by dual anti-viral and immunomodulatory mode of action[10]. In contrast to nucleoside analogues, pegylated interferon alpha-2a has a higher rate of HBeAg seroconversion, lower rate of relapse after treatment cessation, moreover, it has not been observed to induce mutation of HBV [11,12]. However, it has a few adverse events such as transient flu-like symptoms, depression and abnormal blood counts [13,14]. In this study, both leucopenia and thrombocytopenia had been observed in case 3 and case 4, so we adopted sequential therapy with pegasys and baraclude (one kind of nucleoside analogues). The results showed that their leukocyte and platelet counts gradually returned to the baseline levels one month after pegasys was paused and baraclude was administered sequentially, then we continued the administration of pegasys. For the reason that sequential therapy could effectively evade the inherent adverse events of pegasys and meanwhile, nucleoside analogues could keep sustained suppression of HBV during the time when pegasys is paused, so the advantageous efficacy of pegasys could be brought into play as much as possible.

By analyzing above materials, we had observed one phenomenon that the clearance of HBsAg occurred in the 4 patients not very long(1 wk, 18 wks, 1 wk and 6 wks, respectively) after they had achieved HBeAg seroconversion. This may imply that pegylated interferon alpha-2a probably plays an important role in helping clear HBsAg by means of activating the host immune reaction. Additionally, ALT levels in the 4 patients were elevated during treatment but gradually normalized at the end of therapy, they also had no clinical symptoms. Considering that pegylated interferon alpha-2a takes effect by improving the host immune function, we suppose the occurrence of transient elevated ALT might be a reflection of the course during which HBV infectious hepatocytes are being eradicated.

Although limited case numbers are reported in this article and more patients need to be included for further study, one certain clue, which can be provided to clinical doctors, is that some patients with HBeAg-positive CHB could probably be expected to achieve the ideal therapeutic goal of HBeAg seroconversion, HBsAg clearance and even HBsAg seroconversion if they are treated with effective anti-viral drugs and appropriate therapeutic schemes.

## Conclusion

Based on our clinical observation, either pegasys monotherapy or sequential therapy with pegasys and baraclude is efficacious and relatively safe for treating patients with CHB.

## Competing interests

The authors declare that they have no competing interests.

## Authors' contributions

YJ conceived the study and made substantial contributions to its design, acquisition, analysis and interpretation of data. ZL participated in the design and revised the manuscript critically for important intellectual content.
